# The role of exploration and exploitation in primate communication

**DOI:** 10.1098/rspb.2024.1665

**Published:** 2025-01-22

**Authors:** Marlen Fröhlich, Cedric Boeckx, Claudio Tennie

**Affiliations:** ^1^ Paleoanthropology, Institute for Archaeological Sciences, Department of Geosciences, University of Tübingen, Rümelinstrasse 23, Tübingen 72070, Germany; ^2^ DFG Centre for Advanced Studies ‘Words Bones, Genes, Tools’, University of Tübingen, Rümelinstrasse 23, Tübingen 72070, Germany; ^3^ Section of General Linguistics, University of Barcelona, Gran Via de les Corts Catalanes 585, Barcelona 08007, Spain; ^4^ Institute of Complex Systems, University of Barcelona, Gran Via de les Corts Catalanes 585, Barcelona 08007, Spain; ^5^ Institute of Neurosciences, University of Barcelona, Gran Via de les Corts Catalanes 585, Barcelona 08007, Spain; ^6^ Catalan Institute for Research and Advanced Studies (ICREA), Passeig de Lluís Companys 23, Barcelona 08010, Spain; ^7^ Early Prehistory and Quaternary Ecology, Institute for Archaeological Sciences, Department of Geosciences, University of Tübingen, Hölderlinstrasse 12, Tübingen 72074, Germany

**Keywords:** comparative approach, communicative culture, great apes, individual variation, language evolution, social learning

## Abstract

The concepts of social learning and exploration have been central to debates in comparative cognition research. While their roles in the origins of human cumulative culture on the one hand and creativity on the other have been highlighted, the two concepts have mostly been studied separately. In this article, we examine the relationship between adopting similar or different behaviours within a group, focusing on how exploration and exploitation shape primate communication systems. Using a comparative approach, we discuss how similarity and differentiation of communicative behaviour can be viewed as two endpoints on a continuum, impacting both individual- and group-level behavioural variation. While group-level variation is evident in some ape behaviours (e.g. foraging traditions), individual variation in communicative behaviour appears to outweigh group-level differences, making a widespread communicative culture in apes unlikely. Drawing parallels to language acquisition in human infants, we propose that ape communication follows an exploration–exploitation trajectory, with initial exploration gradually giving way to focused exploitation of genetically predisposed and/or individually developed communicative repertoires. By integrating the individual and social learning processes underlying communicative behaviour, we can gain a deeper understanding of how exploration–exploitation tensions shape communication systems across species.

## 1. Individual differentiation and similarity: different ends of the same scale?

Cultural open-endedness and language are two core elements of definitions of contemporary humans, and are often used to distinguish our species from other animals [1]. Cultural evolution, including that of languages, has interacted with behaviour, communication and social structures for thousands of years. The comparative approach, often focused on humans’ close relatives, the great apes, has been widely used to explore the cognitive building blocks of these complex traits and how they are linked. In the past, considerable effort has been made to uncover evidence for communicative cultures of some variants in non-human primates, in particular by using the method of exclusion: the presence and absence of communicative behaviours has been mapped for different populations of great ape species, such as orangutans (*Pongo abelii/pygmaeus*) [2] or chimpanzees (*Pan troglodytes*) [3]. Strikingly, this effort has revealed very little evidence for cultural patterns of communicative variation [4,5] (but see [6]), unlike other great ape behaviours such as tool use associated with extractive foraging [7], and unlike song traditions in non-primate taxa such as cetaceans [8] and birds [9]. Similar to these other behaviours, the communicative domain was found to lack locally unique variants (with a single possible exception, see below) when the method of local restriction was applied to the available datasets and other collected observations [10].

Surprisingly and despite a general lack of evidence for widespread or deep communicative cultures in non-human species, most comparative research has focused on population- or species-level variation but not on communicative variation at the *individual level*. Importantly, primate field studies typically involve relatively small numbers of individuals. Because great apes comprise long-lived species with extended generation times, sample sizes are necessarily quite small. This means that individual differences in signal use, repertoires and contextual/functional specificity (and thus whether a few specific individuals are driving patterns) are typically ignored, dismissed or treated as noise. Accordingly, there is a high probability that single individuals drive apparent group-level phenomena (e.g. by affecting the dynamics of social hierarchies). We still do not know which, if any, communicative patterns in primates reflect stable, repeatable traits (i.e. an individual that shows a particular trait at one time should also show it at later times) [11,12]. More recently, researchers have called for greater effort to look beyond the ‘social group’ level and instead explicitly consider individual-level variation and the many socio-ecological factors that drive it (e.g. [13–15]). Individual differences in signalling can arise in several ways. On the one hand, they can be the result of specific anatomical and physiological constraints along with the acoustic effects of these differences. For example, individual differences in many species (including vocal production learners such as songbirds and humans) can arise from differences in limb movements or vocal tract and organ morphology [16]. This mechanism of generating both group-level similarity and individual-level variation does not rely on any kind of learning. On the other hand, individual differences could also be the result of learning through exploration or observation.

Here, we aim to go beyond the current debate around the role(s) of culture(s) in great ape communication systems. Social interactions undeniably drive variation among individuals, whether through social niche specialization [17], resource competition [18], spatial cohesion [19] or indirect effects [20], potentially making behaviour more similar or divergent [21,22]. However, although documented across a wide range of animal species, behaviours and contexts, *similarity* and *differentiation* are typically considered separately. Recently, it has been argued that these are not independent concepts, but can be integrated into a single scale that considers how social interactions drive changes in inter-individual variance within groups: ‘conformity’ reduces variance within groups, whereas differentiation increases it [22]. However, we believe that the authors’ use of ‘conformity’ is incompatible with the (earlier) definition of the term by psychologists, which refers to the process of aligning one’s attitudes, beliefs and behaviours to match the norms or standards of a group, and has been used since Asch’s classic experiments on conformity [23]. For this reason, and to avoid confusion, we prefer to adopt the term ‘within-group similarity’ for the phenomenon described by Ioannou & Laskowski [22] as ‘conformity’. Given that both individual (e.g. age and sex of interactants) and social factors (e.g. kinship and rank of interactants) affect interactions and thus the communicative repertoires of individuals, we here explore the idea that individual differentiation and similarity at the group level in ape communicative behaviour are also essentially just two ends of a single, continuous scale, and may even to some extent be related to the exploration–exploitation tensions we see in human development and language learning.

This article has three major goals. First, we review the evidence for both individual and social learning processes underlying the acquisition of communicative signals in great apes. Second, we will argue that comparative research would benefit from recognizing that behavioural similarity and differentiation can be placed at different ends of the same scale, each defined by its impact on individual-level (and group-level) behavioural variation [22]. Such a view would allow for a deeper—but at least more practical and economical—understanding of how social interactions drive changes in signal use within groups. Third, we will show that great ape communication may resemble the exploitation–exploration tensions in human language acquisition [24], based on insights by [25,26]. In essence, we recognize that some non-human species, and especially great apes, have an early disposition towards exploration that allows them to learn a wide range of signal features, while their increasing experience leads to a more focused and efficient exploitation of known or intrinsic communicative resources in the adolescent phase. We will also discuss maturational trajectories and consider whether the potential switch to exploiting known (including genetically predisposed) resources would be expected to occur earlier or later than in humans. Such trajectories would also be reflected in the variable differentiation and similarity of communicative patterns across primate groups.

## 2. The role of learning in the communicative behaviour of primates and beyond

While this review focuses largely on great apes, this is by no means to say that they are a prime example for the generation of novel signals or their social transmission. In fact, vocal (social) learners appear to be more prominent among other taxa such as birds, bats, pinnipeds, cetaceans and elephants [27], with the extent of vocal learning reported to range from subtle changes in the structure of species-specific signals (as demonstrated in zebra finches [28]) to extensive repertoires of novel vocalizations (e.g. mockingbirds imitating the songs of other animals [29], parrots imitating human speech [30]). The copying of novel songs is important here because the reproduction of species-typical songs may rely on processes other than copying, sometimes in subtle and not easily detectable ways [31]. We also note that, while not every claim of song-copying excludes potential influences of human training (e.g. many cases of human-raised parrots), some studies have successfully done so experimentally. Such studies—untrained birds, tested for the copying of non-species typical song—have clearly shown that at least some bird species have (apparently spontaneously emerging) abilities to copy novel songs [32]. The classic studies of vervet (*Chlorocebus pygerythrus*) monkey alarm calls showed that juveniles produce distinguishable versions of the three fundamental call types very early in development, but only learn to use them accurately with experience [33]. This allows the calls to ‘shift meaning’ and be applied to different threats in different groups. Similarly, studies on the closely related West African green monkeys (*Chlorocebus sabaeus*) have shown rapid learning to adjust a call to the presence of a drone [34].

We chose to focus largely on great apes to develop an argument about the presence of exploration–exploitation tensions in non-human animal communication for several reasons: first, humans share with great apes the closest phylogenetic relationship, a particularly slow life history and relatively high social complexity [35]. Second, while the vocal repertoire of great apes may not be particularly large, the visual (i.e. facial and gestural) repertoire is exceptionally rich [36,37], and there is evidence for enormous flexibility and learning capacities across all communicative modalities (e.g. [6,38–41]). There is also solid evidence that great apes occasionally invent or individually learn new signals from scratch [42–44], and gestural idiosyncrasies have been reported for all great ape species [45–48]. Third, and most importantly for our account, hominids appear to be the only taxon of long-lived species whose current research record allows us to assess the presence of exploratory behaviour leading to the individual emergence of novel (i.e. species-atypical) signal forms, but also the presence of socially learned signal *use* (and thus the relative contribution of individual versus social learning) [48,49]. Of course, absence of evidence is not evidence of absence. Future work, assisted by the views developed here, can hopefully provide a richer comparative landscape.

## 3. Group-level variation in communicative behaviour

It is well established that apes behave similarly within groups in some domains, most notably tool use (e.g. [50,51]). Given the influential and widely cited reports of geographic variation between sites in the specific mix of ape behavioural patterns [3,52], it might at first seem likely that behaviour-copying social learning mechanisms (e.g. special types of imitation) would have to be involved in the creation and maintenance of these patterns. This is, however, not a logical necessity: a study using agent-based models that lack such detailed copying mechanisms produced similar patterns [53]. While some types of social learning must be relevant to ape cultures, such studies show that patterns of ape culture cannot be used to infer specific social learning mechanisms. Of course, this does not negate the existence of ape culture *per se*. The fact that such signatures can come about in a model without behaviour copying does not prove the absence of all types of social learning. In fact, the best way to interpret the agent-based model just mentioned is that the addition of alternative social learning mechanisms to the model (those that cannot copy behavioural details) was necessary to produce a close approximation to ape cultural patterns observed in the wild. And since culture is essentially equivalent to a social learning effect, this means that ape cultures are very likely to be real. Empirical evidence from the wild likewise supports this conclusion: even neighbouring ape communities, despite sharing much of their genetic and environmental make-up, have been found to express different mixtures of behaviour [54]. The best explanation (by exclusion) is that some social learning mechanisms were at play. *Ergo*, culture was at play.

What all this means for ape communication is the following. Whether social learning transmits the behaviour itself (e.g. via imitation) or stabilizes certain behaviours more than others, these processes would all lead to communicative differences between ape populations if ape communication were affected similarly to ape behaviour in other domains (e.g. tool use). When genetic similarity is controlled for, greater similarity of signal repertoires within groups than between groups would have provided evidence for such social learning processes [55]. The existence of more than a few group-specific, locally unique communication behaviours would even have made behaviour-copying variants of social learning more likely [10]. So, do we see evidence for a communication culture in apes similar to that seen in other domains [3,52]? Looking at the same species, there is at best partial, weak evidence for communicative variation between study groups. In captivity, chimpanzees use several invented, attention-getting sounds or vocalizations directed at human caretakers [43], the frequency of which is likely to be influenced by social learning from mother to offspring [56]. Several alleged locally restricted (i.e. not expressed in all social groups of a given species) gestures have been reported for zoo-housed groups of chimpanzees [57], gorillas (*Gorilla gorilla*) [58,59] and orangutans [60]. Such locally restricted signals are even more infrequent in the wild. Most evidence for the invention and subsequent social learning of sounds and vocalizations has been described in wild orangutans, such as the ‘kiss-squeak’ variants (a sound produced in distress, occasionally involving tool use), ‘nest smacks’, ‘nest raspberries’ (both produced during nest building) and ‘throat scrapes’ or ‘harmonic uuhs’ (produced by mothers before retrieving their infant) [2,61]. Of these, the ‘harmonic uuh’ seems to be the only truly novel signal, as it does not occur in any other study population [10]. By looking at more fine-grained features of communicative acts (e.g. detailed body parts or flexion of specific limbs), researchers then aimed to uncover better/more evidence for group-level signatures in gestural and vocal communication. One relevant candidate for a ‘patchy’ cultural signal variant was considered: the ‘leaf clipping’ gesture used by wild chimpanzees, which seems to vary to some extent between wild communities in form and function [62–65]. Similarly, divergence in the sounds performed by chimpanzees during grooming (e.g. ‘splutters’ and ‘teeth chomps’) between populations has been taken as evidence for sound dialects [66]. Among non-ape primates, invented communicative behaviours have been described for several capuchin species, such as the ‘hand-sniffing’, ‘sucking’, ‘finger-in-mouth’ of white-faced capuchins (*Cebus capucinus*) [67], and the ‘stone-throwing display’ performed by proceptive females of bearded capuchins (*Sapajus libidinous*) to solicit copulations from males [68,69].

Other studies have found no support for the notion of dialects in wild populations. A study on the pant-hoot, a common long-distance call used by wild chimpanzees, revealed no statistically significant differences between neighbouring chimpanzee communities (Gombe) or between geographically distant communities. Instead, the data suggested that variation in chimpanzee pant‐hoots reflected individual differences rather than group differences that would indicate dialects in this population [14]. Interestingly, the pant-hoot is used across all chimpanzee communities, many of which differ in specific foraging and other behavioural variants [3]. In addition, a study investigating the onset of gesture production and early use in captive great ape infants showed that bonobo (*Pan paniscus*) and chimpanzee infants share a considerably greater portion of their gestural repertoire with individuals of their peer groups than with their mothers [70]. Both vertical and horizontal social transmission of gestures were ruled out because (i) the same patterns of ‘peer group-specific’ gesture use were found across species and across study groups, (ii) similar use of gestures within age groups may largely result from exposure to the same social contexts [70]. These and other findings show that great ape infants are unlikely to copy their mothers’ gestures, ruling out the possibility of ‘matrisyncratic’ gesture transfer [71]. Moreover, several gesture types exchanged within mother–infant dyads are so-called ‘one-way gestures’, produced by only one member of the dyad (e.g. lowering the back to invite the infant to climb on the mother’s back; [72]). Such signals are therefore even less likely to be copied than others (after all, they look different to each partner). However, it is possible that an infant could copy such a gesture, but only produce it for others of its own size or smaller. To see whether such signals are copied in both form and function, it would be necessary to study the communicative behaviour of female infants directed at their own offspring a decade or so later. Unfortunately, this is largely impossible in a primate species characterized by female dispersal, such as chimpanzees and bonobos.

An important question to ask when studying communication patterns on the group level should be: why expect group-level similarity in signal use (or a lack of individual differentiation) when a diverse communicative tool-set is already available to all individuals in a social group? In other words, why differ from signals that everyone else already uses (and understands!)? The selective advantage of gaining access to a new energy-rich food resource via a suitable tool may be obvious, but why would it make sense to copy another individual’s truly novel communicative signal, regardless of existing alternative efficient signals in my repertoire? While novel behaviours can arise (e.g. via down the line effects of genetic mutation or innovation), these alternative origins of signal form are relatively rare, as most signals are drawn from a shared and latent repertoire [36,46]. From what we know from field and captive research settings, there is also little reason to expect any form of ‘punishment’ or other disadvantages for using different signals, so the only answer could be that some (innovated and copied) signals are proven to be much more effective or efficient than others. This may be plausible for ‘evolutionarily urgent’ [73] gestural signals used by males to solicit copulations or consortship with females (e.g. leaf-clipping?), where it may pay to use mating opportunities efficiently in the presence of competing bystanders. Such an explanation is less plausible for most other social contexts, or for social coordination in general, and indeed we often see remarkable individual variation in the use of gestures to solicit grooming, joint travel or social play [4,55]. Primate communication research has neglected the role of exploration and learning via ‘signal probing’: trying out different signals that may be more or less functional in certain contexts and with certain interaction partners. Moreover, all else being equal, the time to try things out is more likely to occur early in ontogeny rather than later in development. In the following section, we discuss the role that exploration may play in shaping primate behavioural repertoires early in ontogeny.

## 4. Exploratory tendencies in the behaviour of non-human primates

The role of exploration in shaping not only foraging but also social behaviour in non-human species has been increasingly recognized in recent years. Some great apes explore novel objects with no intrinsic value solely to reduce uncertainty and seek information [72]. Social exposure seems to be essential in fostering this active novelty seeking or ‘curiosity’: studies on wild orangutans of two different species (*Pongo abelii/pygmaeus*) have provided evidence that sociability fosters both immediate and later exploratory tendencies [74,75]. In addition, studies on curiosity and problem-solving in orangutans have revealed a striking wild-captive contrast, with captive individuals generally being much less neophobic than their wild counterparts [76,77]. In general, reduced constraints (no predation, no foraging) and increased opportunities (loss of neophobia, behavioural enrichment) in captivity appear to promote innovation [35,78–81]. Notably, this captivity effect [82] concerns not only potentially highly adaptive foraging techniques, but also innovation in the communicative domain [43,83,84]. This is relevant because the same novelty-seeking mechanisms seem to underlie both curiosity and creativity, both being affected by similar cognitive faculties and involving the same brain regions [85]. Accordingly, curiosity is widely regarded as the cornerstone of innovation [77,86]. In summary, comparative evidence suggests that the rise of the exploratory tendency in human evolution seems to lie at the very core of the sharp increase in the diversity and complexity of linguistic and material culture [87].

## 5. The concept of exploration–exploitation tensions

Exploration–exploitation dilemmas are fundamental to decision-making processes in many fields, involving a balance between two competing extreme strategies at the ends of a continuous scale [88]. Exploration involves trying out new options or gathering information that could lead to better outcomes later. This approach can uncover potentially better alternatives, prevents stagnation in suboptimal choices, and often requires taking risks and sacrificing short-term benefits. By contrast, exploitation focuses on selecting the best known option based on current information to maximize immediate returns. This strategy leverages existing knowledge to make optimal decisions, prioritizes short-term gains and is generally considered a safer route that avoids risks [89]. Tension between these strategies arises when decisions yield new information, creating a feedback loop, and achieving the right balance is essential for enhancing long-term performance and adapting to changing circumstances [90]. This trade-off has been shown to be relevant in various fields, including economics, psychology, machine learning and ecology. For instance, in product development, it is the choice between improving existing products or developing new ones [91]; in psychology, it is the choice between allocating resources to tried and tested methods versus exploring new possibilities [92]; and in machine (reinforcement) learning, agents must navigate the choice between exploring new actions and exploiting known successful strategies [93]. Taken together, trading off exploration against exploitation in decision-making is critical for adapting to change, avoiding suboptimal paths, balancing immediate and future rewards, and facilitating effective learning in uncertain environments [88,94].

## 6. Exploration versus exploitation in human development

To better understand the apparent contrast between behavioural differentiation and similarity at the group level in ape communication, it may be worth drawing relevant parallels to the exploration–exploitation tensions identified in human development [24,25]. While both exploration and exploitation are present throughout most stages of human development, children’s natural inclination to explore—likely supported by their heightened cognitive and neural plasticity—is well suited to the challenges of early life. This tendency enables them to learn fundamental aspects of the physical and social world, such as conventions [95]. The strong predisposition for exploration is particularly relevant for long-lived animals, especially humans, who live in diverse socio-ecological and physical environments that offer a wide range of behavioural options [95]. Furthermore, the protracted life history of humans may serve to balance the delicate trade-off between exploration and exploitation by allowing members of the species to both explore extensively during childhood while under the protective care of adults, and to exploit after maturity, with the benefit of updated environmental information provided by a long, protected exploratory phase preceding the exploitation phase. As children grow older and gain more experience, they narrow the possibilities they consider, and become more efficient at exploiting known resources. Moreover, the potential generation of new behaviours can also be adaptive to circumstances that change over the span of a generation. For example, if the social environment changes profoundly over the course of a lifetime due to demographic shifts, the exploratory use of signals will pay off in adapting to such changes without having to rely on the elders of the group.

Importantly, the explorative/creative versus exploitative use of communicative utterances early in life is also a crucial aspect of language learning [24,26,96]. Children’s early disposition towards exploration allows them to discover and learn a wide range of unfamiliar linguistic features (structures that deviate from the input they receive), whereas exploitation involves capitalizing on the knowledge already gained. Human language evolution constantly provides a background against which entirely new variants are created and thus can (and must) be explored. This dilemma can be seen in language learning as a balance between using known vocabulary and grammar to communicate (exploitation) and innovating new words and structures to improve language skills (exploration). Moreover, infants begin with the ability to learn all the phonetic contrasts in the world’s languages [97–99]. As they grow older, the range of possibilities they consider narrows, making it more difficult for them to hear, let alone use, fine-grained distinctions in other languages [100,101]. Their growing experience leads to a more focused and efficient use (exploitation) of known linguistic resources [25]. This early broader search allows more exploration of the potential space, but a more finely tuned system is more efficient later on. A recent study of children’s vocal experiences in Vanuatu showed that the strongest association between children’s own vocalization counts and input counts was with those of other children, not with those of adults [102]. Similarly, child-to-child teaching of subsistence skills was shown to be more common than adult-to-child teaching among hunter-gatherers, highlighting the potential importance of peer cultures [103]. These findings underscore the link between extended childhood and language learning in humans.

## 7. Exploration and exploitation in great ape communication

Comparative research suggests that similar exploration–exploitation phenomena may be present in the development of non-human ape communication systems, suggesting an important layer of underlying continuity. Although non-human primates lack a prolonged childhood, great apes have an exceptionally slow life history within the primate order, with extended juvenile periods and late maturation ages [104]. The importance of great apes’ slow life history is highlighted by the slow development of skills associated with food processing in orangutans [74]. The orangutans of northwest Sumatra are characterized by much higher rates of association, interaction and tool use than their Bornean counterparts [105]. In these Sumatran orangutans, later age at weaning (7.5−9 versus 5−7 years) and later age at first reproduction (14−16 versus 13−14 years; [106]) correspond to later age at skill competence (i.e. adult-level feeding rates for difficult techniques attained at 12−14 versus 10−11 years): growth can be prioritized and completed only after full skill competence is achieved, but adult skill competence is still achieved well before the age of first reproduction.

Because young individuals are more likely to engage in exploratory behaviour in all domains and across animal taxa (e.g. [25,107,108]), this age group may also be more likely to innovate in the communicative domain. The longer developmental period of great apes, like that of humans, may thus lead to (and perhaps also result from the need for) an extended exploratory period, making signal innovation—and thus individual differences—more likely. In general, individual differentiation of signals relative to group members and age at maturity appear to be correlated in primates [109,110] and other long-lived mammals (e.g. cetaceans: [111]), but systematic cross-species data are, to our knowledge, lacking. The difference between exploration and exploitation in great ape communication lies in the balance between trying out new signalling elements to improve skills (exploration in the form of communicative innovation) and using existing knowledge for effective communication (exploitation of a genetically predisposed repertoire). At least on the basis of what we currently know about ape gestural communication systems, ontogeny may indeed echo phylogeny.

First, there is good evidence for a profound impact of exploration on early communicative development and repertoires in great apes [49]. In infant chimpanzees, the use of 'rapid-fire' sequences of gestures remains frequent until around 4 years of age, when about half of the gestures are produced within rapid series of multiple gestures without pauses. This proportion decreases steadily until adolescence, i.e. after around 10 years of age [110]. Similarly, the use of strings of gestures to persist in communication to achieve their social goals (gestural ‘bouts’ that include sequences of the same or different signals separated by pauses with response waiting) first increases during infancy, peaks in juvenile individuals and then decreases again with maturity [110,112]. The gestures of older individuals were more likely to be successful, and highly successful gestures were often used alone. From these findings, Hobaiter & Byrne [110] concluded that the rapid succession of gestures may be a mechanism by which young apes explore their relatively large repertoires and learn to deploy the most efficient signals (‘repertoire tuning’). Since these efficient signals can vary widely between individuals, each individual may enter adolescence with their own unique communicative repertoire, even though the overall repertoire is highly similar across populations. Whether this phase is akin to a critical period in language learning remains to be investigated.

Second, regardless of age group, gesture types used are shared similarly with individuals in the same social group and with individuals in different social groups, with the amount of sharing being relatively low in both cases [60,113–115]. The use of idiosyncratic signals (i.e. signals that are unique to specific individuals and thus not cultural) appears to occasionally emerge in all great ape (e.g. [113,116–118]) and several monkey species (e.g. [47,119]) across age groups, even with large sampling efforts, whereas group- or matriline-specific gesture types are largely absent [110,120]. Indeed, we know of only one case (a vocal ‘harmonic uuh’) of truly group- and species-specific and thus locally unique signal in all ape communication [10]. Some authors interpret these findings as indirect arguments for the ontogenetic ritualization [57,116] or social negotiation hypotheses [4,121], positing that individual-interactional learning processes rather than imitation underlie the acquisition of gestural signals. Whatever the true underlying cause, these data indeed speak against any behaviour-copying interpretation (be it imitation for gestures or emulation/reproduction of physical sounds for vocalizations).

Third, we know that under specific social and environmental conditions, great apes can supplement their species-specific repertoire with certain signals acquired during their lifetimes. Captive settings are known to trigger exploratory and innovative use of both tools and communicative signals. Conditions such as increased terrestriality, food provisioning and absence of predators may ultimately lead to higher rates of innovation [35,78–81]. Because captive primates experience very different environments from wild primates, the types of events that it would be useful to signal about are also very different, which could trigger the emergence of novel signals. Thus, signal innovation could be driven by both opportunity (free time and energy that would otherwise be spent on survival-related behaviours [80]) and necessity: human-modified, artificial habitats that separate individuals may also require the innovation or modification of signals that are not needed in the wild [48]. In line with this premise, studies on wild-captive contrasts in orangutan communication [83,122] have argued that some signal types used primarily by juvenile or adolescent individuals (such as headstands, which are exclusively observed in zoo play situations) may qualify as ‘weak innovations’’ [123]: communicative signals that readily (re-)emerge independently in different individuals when conditions permit [81]. Similarly, 9 out of 68 gestural and vocal signals in hamadryas baboons (*Papio hamadryas*) were reported only in captivity, so this may also apply to other primate species [84]. Another possibility is that these signals emerged and were then copied by others (but the lack of group-specific variants speaks against this possibility).

We emphasize that the captivity effect discussed above and prolonged development may act as opposing forces (because captive apes are thought to develop more rapidly, with earlier age at first reproduction, due to abundant and reliable food), and that this dynamic is worthy of future study.

In summary, great apes’ extended developmental periods promote exploratory communicative behaviour, with young apes experimenting with gestures and gradually refining their communication skills. This leads to individual-, rather than group-specific, signal repertoires, suggesting that learning processes are driven by interaction rather than imitation. In captivity, where apes experience altered conditions, innovation in communicative signals is more likely, driven by necessity and opportunity. However, captivity may also accelerate development due to resource abundance. Overall, the balance between exploration and exploitation in communication reflects the impact of ontogeny and environmental context on great ape signalling.

## 8. Why is there so much differentiation but so little similarity at the group level in ape communication?

Given the growing evidence for latent innovative potential in non-human ape communication, a central question must be: why are there so many reports of innovated signals, but so few hints of subsequent social learning? Some have pointed to the potential for communicative culture in non-human species [124], but the evidence remains scarce and mostly limited to a few specific signal variants used in specific contexts (e.g. the infant-directed ‘throat scrape’ and ‘harmonic uhh’ calls of orangutan mothers [2] are among the most compelling examples) or ‘dialects’ (variation in the structure or contextual use of specific widely observed gesture types [65,125]). We argue that this paucity of evidence for signal traditions, particularly in the gestural domain, may be related to their limited functional value or ‘evolutionary urgency’. By adopting a new extractive foraging technique, triggered and socially mediated by conspecifics, one can gain access to entirely new, nutrient-rich food sources. In many cases, this is different for communicative behaviour: the use of specific signals may not be very consequential in most cases. To explore this idea, we have to assume (counterfactually) that apes are naturally very good at copying behaviour. Then, if I as an ape do not use this particular signal that I have observed in others, negative consequences will be unlikely (note that alarm calls would have to be an exception here; these are almost certainly genetically predisposed in their form, but probably not in their meaning or ‘correct use’ [33]). I can simply use another signal—either from my species-specific repertoire that has or has not been expressed before (perhaps due to environmental constraints) or, if not available, create an entirely new one for which naive recipients (over repeated instances of interaction) infer meaning from context and initial responses. Great ape communication is extraordinarily variable and flexible: if I cannot obtain my social goal using a given signal, there are usually several other communicative means to obtain it, and often also non-communicative means (e.g. a chimpanzee mother can initiate joint travel with her infant either by signalling or by physical action, [113]).

Of course, there is also the possibility that we have not yet looked at the right signal components, combinations, research settings and species. Many communicative signals are inconspicuous compared with other behaviours and are easily overlooked in a general observational research protocol, especially when we think of low-frequency sounds or silent gestures used in close-range communication (e.g. [126,127]), so chances are that we have missed some cultural variants. Indeed, recent efforts have been made to look at ape communication ‘under the microscope’. A bottom-up approach to analysing communicative behaviour may help to rule out this explanation in the future [128]. Nonetheless, current data strongly suggest that the observation and subsequent imitation of conspecifics’ communication plays a smaller role in communication than has been suggested for other behaviours.

## 9. Individual- and group-level variation: two ends of a single scale

By definition, most communicative acts involve at least two individuals and are used to navigate social interactions. What has been referred to as ‘individual learning’ for the acquisition of communicative signals (e.g. [115]) is therefore in some sense not equivalent to individual learning where only one individual is actually acting alone. This was demonstrated, for example, by the innovation of nut-cracking skills in nut-cracking-naive captive orangutans tested in isolation [129]. When a signal is directed to one or more recipients at close range, social interaction is usually taking place. Thus, some extent of ‘social learning’—at least in the broadest sense of the term—can never be ruled out. Of course, this does not mean that all these actions will or must lead to signal copying (indeed, they usually do not; see above). Yet, the situation is social, and something can be learned (e.g. the meaning that is inferred) in a broad sense because of this social setting. Importantly, since social learning can involve directing attention through the observation of another individual’s behaviour, it can also be the relevance of a particular stimulus or set of stimuli that is transmitted and learned.

**Figure 1.**
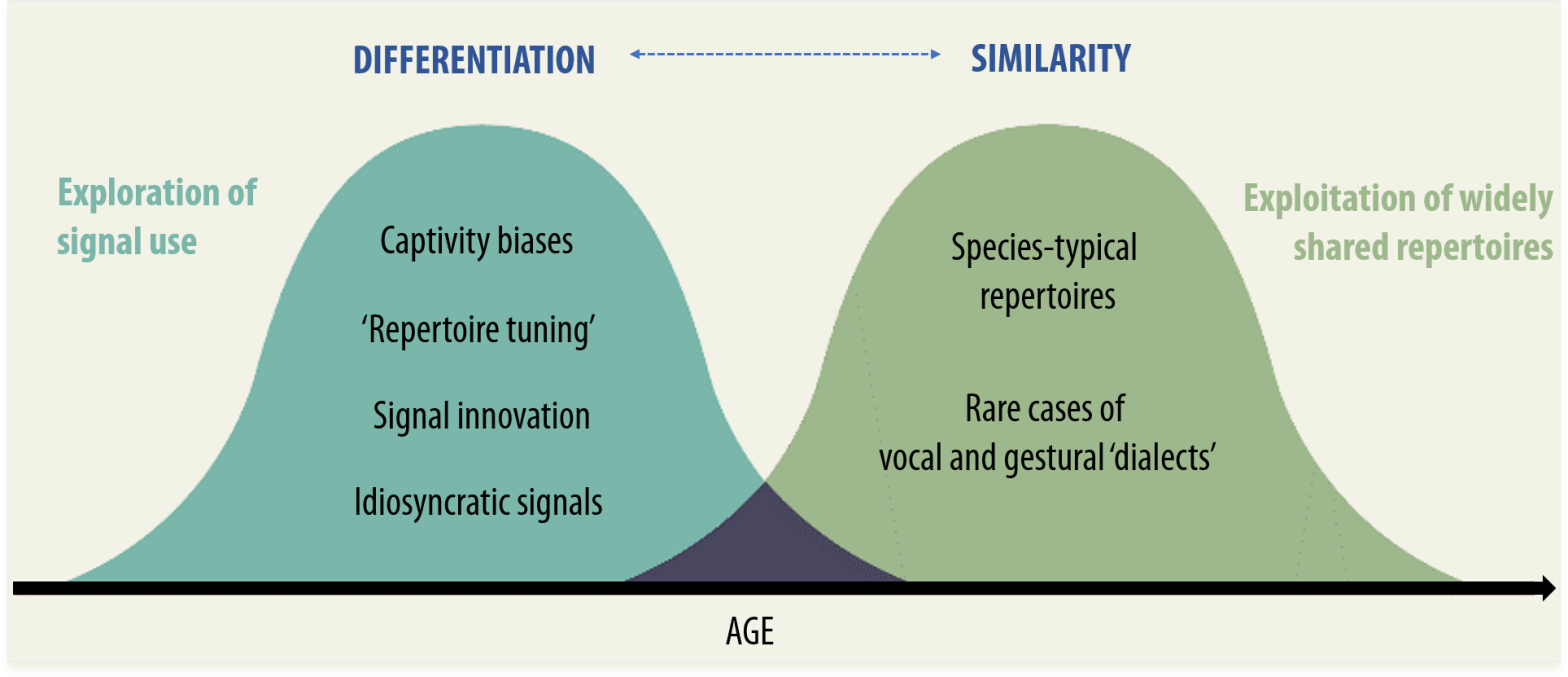
Evidence of individual differentiation reflecting exploration and similarity reflecting exploitation in developmental trajectories of great ape communication.

According to both ontogenetic ritualization [115] and social negotiation [4,113], communicative signals can even be *shaped* by repeated social interactions with the same and different individuals. Again, shaping need not involve copying (in some sense, even a nut shapes its cracking behaviour). However, the forms of the signals can be affected in both cases. Moreover, the new ‘recruitment view’ posits that even if alternative signal forms are socially learned, their interpretability is likely to be driven by the presence of easily interpretable bodily states [46], even if the particular signal forms vary between populations (e.g. leaf-modifying gestures across chimpanzee communities, [65]). In sum, all these acquisition mechanisms by definition have a strong interactional component, even though the signal forms are most likely neither imitated (gestures) nor emulated (sounds). Therefore, both similarity and differentiation may be present to varying degrees in great ape populations, depending on the species and the behaviour being studied (figure 1). Similar to what has been discussed for the extent of individual variability in non-communicative behaviours [22], comparative research may benefit conceptually from placing individual and social learning of communicative behaviour at different ends of the same scale. This will allow a deeper understanding of the relationship between social interactions and inter-individual variation. Only by decomposing behavioural variation into its individual- and group-related components can we demonstrate that an increase in group-level variance indicates that individuals within groups converged in their behaviour to generate group divergence. An important avenue for future research will be to assess the relationship between exploratory behaviour and shared repertoires in other long-lived species, such as whales or elephants, to see whether similar exploration–exploitation tensions could convergently arise.

## 10. Conclusions

Understanding the factors shaping great ape communication requires a nuanced approach that takes into account both individual- and group-level variation. While evidence for widespread communicative cultures in non-human primates remains limited, this does not negate the role of social learning and individual differentiation in shaping their communicative behaviours. The exploration–exploitation dynamics observed in human (language) development offer a useful parallel for considering how these processes might manifest in great apes. Moreover, researchers can gain deeper insights into the underlying mechanisms driving communicative behaviour by focusing on the balance between similarity and differentiation within social groups. Future research should explore the fine-grained aspects of ape communication and the socio-ecological factors that drive individual versus group differences, potentially also revealing more about the evolutionary pathways of humans’ linguistic capabilities.

## Data Availability

There is no data and source code to report for this manuscript.
